# Assessment of attitudes towards antihypertensive medication among Hungarian patients with hypertension using the beliefs about medicines questionnaire: a validation and cross-sectional study

**DOI:** 10.1186/s12889-026-26559-2

**Published:** 2026-02-07

**Authors:** Mihály Varga, Klára Bíró, Viktor Dombrádi, Nóra Kovács, Attila Nagy, Gábor Bányai, Klára Boruzs

**Affiliations:** 1 Doctoral School of Health Sciences, University of Debrecen, Debrecen, Hungary; 2 Institute of Health Economics and Management, Faculty of Economics and Business, University of Debrecen, Debrecen, Hungary; 3 Department of Public Health and Epidemiology, Faculty of Medicine, University of Debrecen, Debrecen, Hungary; 4 Department of Health Informatics, Institute of Health Sciences, Faculty of Health Sciences, University of Debrecen, Debrecen, Hungary

**Keywords:** Medications, Attitude, Belief, Hypertension, Patiens, BMQ

## Abstract

**Background:**

Assessing the beliefs of hypertensive patients about medications is crucial for appropriate and preventive drug treatments. The key to effective hypertension care is the patient’s proper cooperation, which is why it is important to examine their beliefs regarding antihypertension medication use. The aims of the study were to validate the Hungarian translation of the Beliefs about Medicines Questionnaire (BMQ) and to identify possible factors that might influence the attitude of patients with hypertension towards medications.

**Methods:**

Data was collected in Hungary using the BMQ. 1,067 adult patients with chronic hypertension and taking their prescribed medications took part in the research. Statistical analysis was done to validate the questionnaire and to identify which sociodemographic factors influence the different aspects of attitudes toward medication.

**Results:**

The translation showed good reliability and validity. Furthermore, respondents who worked in healthcare were more likely to feel the need to take antihypertensive medications than those who did not (*p* < 0.001). Also, participants who reported good or very good (*p* < 0.001) and fair (*p* = 0.021) financial status were more concerned about antihypertensive treatment than those who reported poor or very poor financial status. Of those respondents, who answered living in towns of less than 1,000 people were more likely to believe that antihypertensive treatment was harmful compared to those living in towns of more than 100,000 people (*p* = 0.007).

**Conclusions:**

For the identified groups, more attention is needed to ensure that the medication is taken correctly. This requires the promotion of good doctor-patient communication.

## Background

### Hypertension treatment

Early recognition of the disease and appropriate treatment, which may involve the use of medication, would be of great importance for patients suffering from hypertension. One of the keys to effective patient care is good communication and cooperation between the patient and the doctor [1], therefore it is important to investigate the patients’ medication use and adherence.

Between 1990 and 2019, the number of people with high blood pressure (those with blood pressure 140/90 mmHg or higher or who are taking antihypertensive medications) doubled from 650 million to 1.3 billion [2]. Today, nearly half of the world’s hypertensive patients are unaware of their condition and more than three-quarters of adults with hypertension live in low- and middle-income countries [2, 3]. 

Prevention, early detection, and effective treatment of hypertension is one of the most cost-effective healthcare interventions and should be a priority for countries as part of the national healthcare package at the primary care level. The economic benefits of improved hypertension management programmes are high [2]. 

Over the past 20 years, the number of patients in Hungary has constantly increased by 1–2% annually, and in 2019 about four-tenths of people aged 19 years and older suffered from hypertension [3, 4]. The other Visegrad countries – namely Czechia, Slovakia and Poland – also have high prevalence (22–26%). The risk of those diagnosed by a physician increases with age, with 16% of those aged 35–44 suffering from this condition [4, 5]. 

The increasing prevalence of hypertension worldwide is influenced by a complex interplay of social, economic, demographic, environmental and genetic factors [6], while its associated risk factors, such as obesity, unhealthy diet and extensive physical inactivity together cause a further increase in the number of patients [7, 8]. One of the key factors in drug treatment is adherence, defined by the World Health Organization as “the degree to which a person’s behaviour is consistent with the recommendations accepted by the healthcare provider” [9]. Several factors, such as patients’ gender, beliefs about medication, disease duration, and education have been identified to be associated with medication non-adherence in patients with various chronic diseases, including hypertension [10–12]. 

Although patients’ attitudes toward medications are often discussed in relation to adherence, these are distinct constructs. Adherence refers to the extent to which patients follow the treatment recommendations provided by their healthcare professionals, while attitudes and beliefs reflect patients’ cognitive and emotional evaluations of their medication (for example perceived necessity, concerns, or trust in therapy). Importantly, attitudes may influence, but do not determine, adherence. As Horne and colleagues emphasized, the Beliefs about Medicines Questionnaire (BMQ) was originally designed to capture these underlying beliefs, independent of behavioral adherence outcomes [13, 14]. In line with this, the current study does not measure adherence or its determinants; rather, it focuses on how sociodemographic characteristics shape patients’ attitudes toward antihypertensive medications.

Understanding the attitudes and beliefs of patients with chronic diseases is essential for effective long-term therapy. Chronic conditions such as hypertension often require lifelong medication, and patients’ perceptions about these treatments strongly influence how they engage with their care. Negative attitudes, such as fear of side effects or distrust in medications may lead to doubts and resistance, while positive attitudes can foster acceptance and cooperation. Previous studies have shown that patients’ beliefs about their medicines provide valuable insights into how they evaluate treatment, and that these beliefs can vary across populations and healthcare systems [1, 13, 14]. Therefore, systematically assessing these attitudes is an important step toward improving patient-centered care and communication in chronic disease management.

### Beliefs about Medicines Questionnaire (BMQ)

To measure medication adherence the Beliefs About Medicines Questionnaire (BMQ) was first introduced by Horne et al. in 1999 and was originally developed in English. It contains two short scales: BMQ-General and BMQ-Specific [13]. The BMQ has been translated into several languages.

There have been several studies investigating the relationship between BMQ data and changes in adherence to medication [15]. The questionnaire has also been used among patients with various chronic conditions, including high cholesterol [15], diabetes [16], chronic obstructive pulmonary disease (COPD) [17], hypertension [18], psoriasis [19], rheumatoid arthritis [20], and asthma [21]. In each case, the BMQ was suitable to answer the research question of each analysis.

### Aims

Thus far, only the BMQ-Specific has been validated in Hungarian [15]. Therefore, the first aim of this study was to validate the entire BMQ for hypertension medication. The second aim was to investigate which sociodemographic factors might influence the opinions of patients taking antihypertensive medication in Hungary.

## Methods

### Study design and sample size

Ethical approval was obtained from The Scientific Research and Ethics Committee of the Medical Research Council (BMEÜ/2801-3/2022/EKU) in Hungary. Data collection took place in 2022 and 2023 among patients suffering from hypertension in Hungary who were taking some type of antihypertensive medication. The data collection was carried out by SZLEM Service L.P. in Hungary. During the study procedure, the participants signed an informed consent form containing all the details of the study. The researcher explained to the patients the aims of the study and assured them that participation was voluntary. No incentives were provided. The participants were asked using an online form, which took about 15 min to fill out. The answers are completely anonymous to all authors of this study. For the data collection market research proprietary panels were used (a total panel size of *n* = 80,000). The respondents were randomly selected and invited to the study from the preregistered panel members.

The aim of the sampling was to get valid answers from *n* = 1,000 hypertension patients, finally we received *n* = 1,067 completed questionnaires out of the 4,000 invitations. This final sample represents the Hungarian hypertensive patient population at a highly reliably statistical level. Participants continuously filled out the questionnaire, and new requests were sent out depending on representativeness. Aftering reaching a 1,000 responses, additional respondents were contacted to ensure representativeness. Thus, the number of participants increased to 1,067. The survey took into account representativeness in terms of age, sex, regions, and the size of the municipalities at a national level. The criteria for participating in this research was being 18 years of age and taking some form of antihypertensive medication. Only those qualified in the participation who answered “yes” to both screening questions. Other exclusion criteria was if not all questions were answered (interrupted the completion).

### Measures

The Beliefs About Medicines Questionnaire (BMQ) was used in this study [13, 14], to assess both the positive and negative beliefs of the participants about medications [22]. 

The Hungarian translation of the BMQ-Specific was previously used to assess attitude towards cholesterol-lowering medication [15]. The original English version [13, 23] was translated into Hungarian by two independent translators. Once completed, the two translations were merged into a single translation and modified so that the questions focused on antihypertensive medications. After that, as part of the validation process, each translation was tested by ten Hungarian-speaking citizens taking antihypertensive medication, and the translations were revised based on their feedback. A third independent translator translated the questionnaire back into English. The back translation was deemed adequate by all members of the research team.

The BMQ consists of 18 questions that assess general beliefs about pharmacotherapy (BMQ-General), and perceptions about pharmacotherapy in more specific situations such as having a chronic illness (BMQ-Specific). The BMQ-General has 8 items [14] and is divided into two subscales. The General-Harm subscale assesses beliefs about the harmful effects of medications, and the General-Overuse subscale addresses the notion that physicians place too much trust in medications [14]. The BMQ-Specific consists of 11 items and is also divided into two subscales. The Specific-Concerns subscale assesses the likelihood of side effects as a result of taking the prescribed medication. The Specific-Needs subscale examines the patient’s belief that he or she must personally adhere to the prescribed medication [14]. Each question of the BMQ has five possible responses ranging from ‘strongly disagree’ to ‘strongly agree’ (from 1 = strongly disagree to 5 = strongly agree). Depending on the distribution of the data, the scores of all subscales are determined by the mean or median of the items.

Higher scores on the General-Harm subscale and the General-Overuse subscale indicate a generally negative attitude toward drug therapy. Similarly, higher scores on the Specific-Concerns subscale indicate the belief that side effects of regular medication use can be harmful, and higher scores on the Specific-Needs subscale indicate that patients need to adhere to their medication regimen to maintain their health [14]. 

The following socio-demographic data were also collected alongside the BMQ: age, sex, highest education level, county, size of municipality, marital status, self-perceived financial situation, self-perceived health status and being a healthcare worker.

### Data analysis

Categorical variables were expressed as frequency (%), while continuous variables were expressed as mean and standard deviation (SD). Cronbach’s alpha was calculated to evaluate the reliability of Necessity, Concern, Harm and Overuse subscales. Internal reliability was considered acceptable if the alpha value was 0.70 or greater [24]. 

As a preliminary analysis, patients were divided into groups based on their beliefs about medication; [8, 25] these groups were created by splitting the BMQ Necessity and Concerns scores at the median. Four categories were created using this method: “skeptical”, “ambivalent”, “indifferent” and “accepting”. Respondents in the “indifferent” category are neither convinced of the need for nor concerned about antihypertensive medications, while “ambivalent” about antihypertensive medications means that the respondent agrees with the need for antihypertensive medications but is also concerned about their possible side effects. “Accepting” antihypertensive medications means that respondents accept the necessity of antihypertensive medications but have low concerns about their possible adverse effects. Respondents who agreed with low necessity and high concern about antihypertensive medications were categorised as ”skeptical”. They have doubts about their personal need and high concerns about taking antihypertensive medications.

Robust regression analysis was performed to evaluate the association between sociodemographic variables and the four subscales. The results were presented as coefficients and the corresponding 95% confidence intervals (CI). Statistical significance was set at a *p*-value of less than 0.05. A confirmatory factor analysis was performed using the chi-square (X^2^), ratio of chi-square to degrees of freedom (X^2^/df), comparative fit index (CFI), Tucker-Lewis index (TLI), root mean square of standardized root mean square (SRMR) and root mean square error of approximation (RMSEA) methods. According to the literature, the recommended values for the X^2^/df ratio are less than 3 [26], the values for CFI and TLI are greater than 0.90 [27], SRMR is less than 0.08, and for RMSEA, lower values such as 0.08 [28] are considered acceptable and values less than 0.10 are only slightly acceptable [29]. When evaluating factor loadings, 0.4 was set as the minimum acceptable value [30]. The STATA v13 (Stata Corp LLC, College Station, TX, USA) software was used to analyse the data.

## Results

### Sample characteristics

A total of 1,067 patients with chronic hypertension taking antihypertensive medications participated in the study. The response rate was around 75%. The average age of the respondents was 57.3 ± 14.1. More than half of the participants were female (63.5%), and most of the participants had a high school education (59.8%). The respondents were classified based on their assessment of their financial situation: very good, good (17.3%), acceptable (61.6%), bad, and very bad (20.9%). The respondents were also categorized by the level of the Nomenclature of territorial units for statistics (NUTS 2) regions of Hungary, and on the basis of the population size of municipality in which they live (Table 1).

**Table 1:** Demographic data of the respondents

	Categories	Mean	SD
Age		57.3	14.1
		**N**	**%**
Sex	Male	390	36.5%
	Female	677	63.5%
Highest education	Primary school	30	2.8%
	High school	638	59.8%
	College or university	399	37.4%
Region (NUTS 2)	Pest/Budapest	359	33.7%
	Central Transdanubia	111	10.4%
	Western Transdanubia	78	7.3%
	Southern Transdanubia	97	9.1%
	Northern Hungary	117	11.0%
	Northern Great Plain	164	15.3%
	Southern Great Plain	141	13.2%
Town size (number of citizens)	1,000–1,999	103	9.7%
	2,000 -4,999	121	11.3%
	5,000–19,999	196	18.4%
	20,000–99,999	269	25.2%
	≥ 100,000	378	35.4%
Marital status	Married or in relationship	661	38.1%
	Other	406	61.9%
Perceived financial situation	Good or very good	182	17.4%
	Fair	646	61.6%
	Bad or very bad	220	21.0%
Healthcare worker	Yes	94	8.8%
	No	973	91.2%
Perceived health status	Good or better	708	66.4%
	Fair or poor	359	33.6%

Table 2 shows the descriptive analysis of BMQ-General items. The Cronbach’s alpha values were 0.74 (Overuse) and 0.83 (Harm). Among the Harm questions in the BMQ-General scale, 40% of the respondents said that “medicines are addictive”. When asked about overuse, the majority agreed with the answer that “doctors prescribe too many medications” (59%) and “doctors place too much trust on medicines” (59%).

**Table 2:** Patients endorsing (Strongly agree/Agree) each overuse and harm statements of the BMQ

Question number	Questions	Strongly agree/Agree			
		*N*	%	Mean	SD
	**Overuse subscale (3 items)**				
1	Doctors prescribe too many medicines.	625	59%	3.61	1.03
7	Doctors place too much trust on medicines.	625	59%	3.61	0.98
8	If doctors spent more time with patients, they would prescribe fewer medicines.	603	57%	3.55	1.06
Total				**3.59**	**0.83**
	**Harm subscale (5 items)**				
2	People who take medication should stop their treatment for a period of time once in a while.	363	34%	3.05	1.06
3	Most of the medicines are addictive.	425	40%	3.17	1.07
4	Natural remedies are safer than medicines.	376	35%	3.18	1.04
5	Medicines do more harm than good.	191	18%	2.72	0.97
6	All medicines are toxic.	343	32%	2.92	1.16
Total				**3.01**	**0.82**

*SD* Standard deviation

The descriptive analysis of the BMQ-Specific items is shown in Table 3. The Cronbach’s alpha values were 0.86 (Necessity) and 0.85 (Concern). Among the Necessity questions of the BMQ-Specific scale, most of the hypertensive patients agreed with the question “I would be very sick without my medication” (64%). In the case of patients with high blood pressure, the most common reason for regularly taking or not taking medication was forgetfulness or a deliberate reduction in the amount of prescribed medication among the participants. Among the concerns related to medications, for the group, the respondents were mostly worried about the long-term effects of the medications (52%).

**Table 3:** Patients rejecting (Strongly disagree/Disagree/Uncertain) each necessity and concern statements of the BMQ

Question number	Questions	Strongly disagree/Disagree/Uncertain			
		*N*	%	Mean	SD
Necessity subscale (5 items)					
1	My health, at present, depends on my antihypertensive medicines.	500	47%	3.46	1.06
3	My life would be impossible without my antihypertensive medicines.	594	56%	3.30	1.11
5	Without my antihypertensive medicines I would be very ill.	679	64%	3.16	1.06
7	My health in the future will depend on my antihypertensive medicines.	618	58%	3.26	1.04
10	My antihypertensive medicines protect me from becoming worse.	305	29%	3.85	0.84
Total				**3.41**	**0.82**
Concern subscale (6 items)					
2	Having to take antihypertensive medicines worries me.	556	52%	3.32	1.21
4	I sometimes worry about the long–term effects of my antihypertensive medicines.	559	52%	3.38	1.16
6	My antihypertensive medicines are a mystery to me.	330	31%	2.91	1.14
8	My antihypertensive medicines disrupt my life.	204	19%	2.43	1.11
9	I sometimes worry about becoming too dependent on my antihypertensive medicines.	369	35%	2.84	1.24
11	These antihypertensive medicines give me unpleasant side effects.	226	21%	2.66	1.07
Total				**2.92**	**0.87**

*SD* Standard deviation

In the case of patients with high blood pressure, the most common reason for regularly taking or not taking medication was forgetfulness or a deliberate reduction in the amount of prescribed medication among the participants.

### Comparisons of the model

Confirmatory factor analysis shows that the fit of the model was satisfactory for both scales (Table 4). Tables 5 and 6 contain the results of the exploratory factor analysis of the BMQ-General and BMQ-Specific. Both scales showed an acceptable level of consistency between the items, as the value of more questions fell below the acceptable threshold of 0.4 [30]. Although, it is worth highlighting that according to the model, two items within the Harm subscale should be a part of the Overuse subscale.

**Table 4:** Fit statistics for BMQ-General and BMQ-Specific scales

Fit Measure	General	Specific
	Two-factor (8 items)	Two-factor (11 items)
X^2^	239.10	241.25
*p*-value	*p* < 0.001	*p* < 0.001
df	19	43
X^2^/df	12.58	5.61
CFI	0.940	0.959
TLI	0.910	0.948
SRMR	0.040	0.042
RMSEA (90% CI)	0.100 (0.09–0.12)	0.066 (0.058–0.074)
*p*-value (RMSEA < 0.05)	*p* < 0.001	*p* = 0.001

*CFI* Comparative Fit Index, *TLI* Tucker-Lewis Index, *SRMR* Standardized Root Mean Square Residual, *RMSEA* Root Mean Square Error of Approximation, *CI *Confidence Interval

**Table 5:** Exploratory factor analysis of the BMQ-General

Item	Hypertension	
	Factor 1	Factor 2
1	0.6460*	0.2469
7	0.6360*	0.2686
8	0.6030*	0.2525
2	0.4934	0.4220**
3	0.4757	0.4310**
4	0.3777	0.4780
5	0.2403	0.8410
6	0.3266	0.6070

* indicates the scales in which a specific item can be found

**shows the items where it is indicated, that it should be part of another scale

**Table 6:** Exploratory factor analysis of the BMQ-Specific

Item	Hypertension	
	Factor 1	Factor 2
1	0.0866	0.7005*
3	0.0981	0.8015*
5	0.1999	0.7959*
7	0.2212	0.7757*
10	-0.0388	0.5984*
2	0.669*	0.1116
4	0.6822*	0.1025
6	0.6584*	0.0985
8	0.754*	0.1237
9	0.7055*	0.0785
11	0.6803*	0.0402

* indicates the scales in which a specific item can be found

### Attitude analysis

**Fig. 1:**
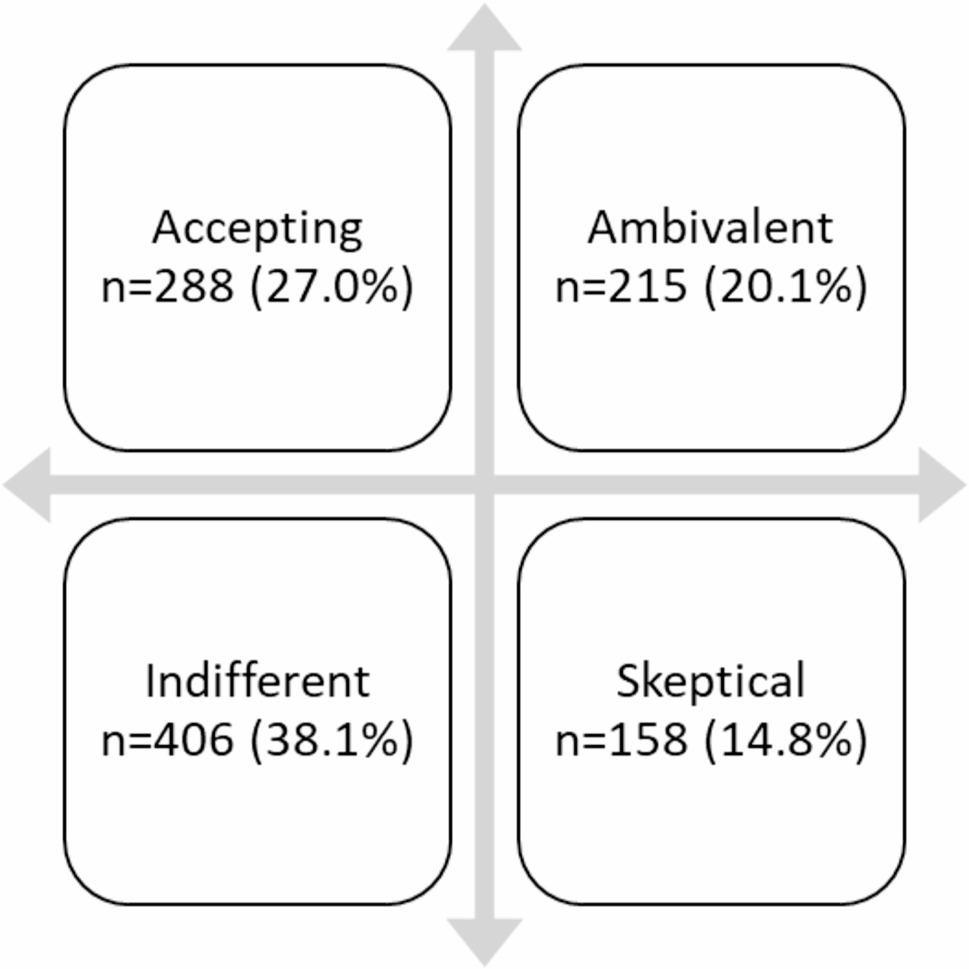
Proportion of the respondents allocated to each attitudinal group regarding antihypertensive medication in Hungary

Patients were classified into “attitude groups” based on their beliefs about antihypertensive medications [31]; these groups were created by dividing the median BMQ-Specific Necessity and Concern scores (Fig. 1). The group that was “skeptical” accounted for 14.8% of the total respondents. 27.0% were “accepting” of antihypertensive medications, while the “ambivalent” group consisted one-fifth (20.1%) of the participants The most common type of patient was “indifferent” (38.1%). 

### Comparative analysis of the responses

The results of the multivariate robust regression analysis for the Harm and Overuse subscales are presented in Table 7. Participants who reported a good or very good financial situation were more likely to think that antihypertensive medications were harmful than those who reported a poor or very poor financial situation (*p* = 0.007). Respondents who worked in healthcare compared to those who did not, were more likely to think that taking antihypertensive medication is harmful (*p* = 0.003). Among participants, those living in municipalities of less than 1,000 people were more likely to think that antihypertensive treatment was harmful compared to those living in towns of more than 100,000 people (*p* = 0.007). Finally, people living in towns of less than 1,000 inhabitants were much more likely to think that antihypertensive medication was overused compared to those living in towns of more than 100,000 inhabitants (*p* = 0.015).

**Table 7:** Multivariate analysis of the harm and overuse subscales

Factors	Harm				Overuse			
	Coef.	95% CI		*p*-value	Coef.	95% CI		*p*-value
Sex								
Female / Male	0.02	-0.09	0.13	0.742	0.04	-0.07	0.15	0.499
**Age group**								
25–34 / **18–24**	0.47	0.00	0.95	0.051	0.50	-0.01	1.00	0.053
35–44 / **18–24**	0.41	-0.06	0.87	0.087	0.42	-0.07	0.91	0.095
45–54 /**18–24**	0.10	-0.35	0.55	0.665	0.27	-0.21	0.74	0.274
55–65 /**18–24**	0.05	-0.40	0.51	0.813	0.44	-0.04	0.92	0.071
65+ / **18–24**	0.06	-0.39	0.51	0.795	0.42	-0.05	0.89	0.081
**Education**								
Secondary / **Primary**	-0.03	-0.35	0.29	0.870	0.33	-0.01	0.67	0.056
Tertiary / **Primary**	-0.20	-0.53	0.13	0.228	0.19	-0.15	0.54	0.270
**Marital status**								
Married / **Other**	-0.01	-0.12	0.10	0.846	-0.04	-0.15	0.07	0.497
**Self-reported financial status**								
Good or very good / **Bad or very bad**	**-0.25**	**-0.43**	**-0.07**	**0.007**	-0.08	-0.27	0.11	0.399
Fair / **Bad or very bad**	-0.12	-0.26	0.01	0.072	-0.05	-0.19	0.09	0.472
**Healthcare worker**								
Yes / **No**	**-0.28**	**-0.46**	**-0.10**	**0.003**	-0.14	-0.33	0.06	0.168
**Subjective health status**								
Good or better / **Fair or poor**	-0.01	-0.12	0.11	0.893	-0.04	-0.16	0.08	0.465
**Resident population**								
< 1,000 / **100**,**000 or more**	**0.39**	**0.11**	**0.67**	**0.007**	**0.37**	**0.07**	**0.67**	**0.015**
1,000–1,999 / **100**,**000 or more**	0.02	-0.22	0.26	0.854	-0.03	-0.29	0.22	0.802
2000-4,999 / **100**,**000 or more**	-0.02	-0.20	0.17	0.863	0.04	-0.15	0.23	0.694
5,000–19,999 / **100**,**000 or more**	0.00	-0.16	0.15	0.955	-0.12	-0.28	0.05	0.167
20,000–99,999 / **100**,**000 or more**	0.04	-0.11	0.18	0.617	-0.01	-0.16	0.14	0.903
**NUTS 2 regions**								
Central Transdanubia / **Pest/Budapest**	-0.05	-0.24	0.15	0.646	0.00	-0.21	0.20	0.967
Western Transdanubia / **Pest/Budapest**	-0.08	-0.29	0.14	0.478	0.01	-0.21	0.24	0.901
Southern Transdanubia / **Pest/Budapest**	-0.05	-0.25	0.15	0.633	0.07	-0.14	0.28	0.518
Northern Hungary / **Pest/Budapest**	0.10	-0.09	0.29	0.309	0.10	-0.10	0.30	0.312
Northern Great Plain / **Pest/Budapest**	0.09	-0.07	0.25	0.256	0.13	-0.04	0.30	0.125
Southern Great Plain / **Pest/Budapest**	0.07	-0.11	0.24	0.451	0.09	-0.09	0.27	0.330

Reference groups are underlined

The multivariate analysis includes all sociodemographic variables as confounding effects

The values in bold are significant

*Coef* Coefficient, *CI* Confidence interval, *p* significance of statistical test; *p* < 0.05: statistically significant

Table 8 shows the results of multivariate robust regression analysis regarding the Necessity and Concern subscales. Healthcare workers were more likely to feel the need to take antihypertensive medications compared to those working in other area (*p* < 0.001). When sociodemographic variables were considered as confounders, participants who reported being in good or better health were less likely to think that they needed to take antihypertensive treatment than those who reported being in fair or poor health (*p* < 0.001).

**Table 8:** Multivariate analysis of the necessity and concern subscales

Factors	Necessity				Concern			
	Coef.	95% CI		*p*-value	Coef.	95% CI		*p*-value
Sex								
Female/ **Male**	-0.01	-0.12	0.10	0.817	-0.02	-0.14	0.09	0.695
**Age group**								
25–34 / **18–24**	0.17	-0.32	0.65	0.499	0.09	-0.41	0.60	0.725
35–44 / **18–24**	-0.09	-0.56	0.39	0.715	-0.22	-0.71	0.28	0.396
45–54 / **18–24**	0.01	-0.45	0.47	0.957	-0.32	-0.80	0.16	0.193
55–65 / **18–24**	0.00	-0.45	0.46	0.988	**-0.51**	**-0.99**	**-0.02**	**0.040**
65+ / **18–24**	0.17	-0.29	0.62	0.475	-0.31	-0.79	0.17	0.200
**Education level**								
Secondary / **Primary**	0.02	-0.31	0.34	0.918	0.29	-0.05	0.63	0.092
Tertiary / **Primary**	0.03	-0.30	0.36	0.860	0.21	-0.14	0.56	0.239
**Marital status**								
Married / **Other**	0.06	-0.04	0.17	0.245	0.02	-0.09	0,14	0.707
**Self-reported financial status**								
Good or very good / **Bad or very bad**	-0.09	-0.27	0.09	0.342	**-0.36**	**-0.55**	**-0.17**	**< 0.001**
Fair / **Bad or very bad**	-0.11	-0.24	0.03	0.129	**-0.17**	**-0.31**	**-0.03**	**0.021**
**Healthcare worker**								
Yes / **No**	**0.35**	**0.16**	**0.53**	**< 0.001**	0.02	-0.18	0.21	0.857
**Subjective health status**								
Good or better / **Fair or poor**	**-0.32**	**-0.44**	**-0.21**	**< 0.001**	**-0.24**	**-0.37**	**-0.12**	**0.001**
**Resident population**								
< 1,000 / **100**,**000 or more**	-0.02	-0.31	0.26	0.869	0.25	-0.05	0.55	0.108
1,000–1,999 / **100**,**000 or more**	0.10	-0.14	0.35	0.408	0.14	-0.11	0.40	0.274
2,000–4,999 / **100**,**000 or more**	0.00	-0.18	0.19	0.979	0.17	-0.03	0.36	0.092
5,000–19,999 / **100**,**000 or more**	0.03	-0.13	0.19	0.722	-0.01	-0.17	0.16	0.925
20,000–99,999 / **100**,**000 or more**	-0.04	-0.18	0.11	0.636	0.04	-0.11	0.19	0.591
**NUTS 2 regions**								
Central Transdanubia / **Pest/Budapest**	0.16	-0.04	0.36	0.117	-0.04	-0.24	0.17	0.727
Western Transdanubia / **Pest/Budapest**	-0.17	-0.39	0.04	0.115	-0.10	-0.32	0.13	0.398
Southern Transdanubia /**Pest/Budapest**	0.06	-0.14	0.26	0.572	0.02	-0.19	0.23	0.844
Northern Hungary / **Pest/Budapest**	0.12	-0.07	0.31	0.227	0.17	-0.03	0.37	0.097
Northern Great Plain / **Pest/Budapest**	0.01	-0.16	0.17	0.939	**0.22**	**0.05**	**0.40**	**0.010**
Southern Great Plain / **Pest/Budapest**	0.00	-0.18	0.17	0.967	**0.18**	**0.00**	**0.36**	**0.049**

Reference groups are underlined

The multivariate analysis includes all sociodemographic variables as confounding effects

The values in bold are significant

*Coef* Coefficient, *CI* Confidence interval, *p* significance of statistical test; *p* < 0.05: statistically significant

Those between the ages of 55–65 were significantly more concerned about antihypertensive medication than those aged 18–24 (*p* = 0.040). Participants who reported a good or very good (*p* < 0.001) and fair (*p* = 0.021) financial status were more concerned about antihypertensive treatment than those who reported a poor or very poor financial situation. Respondents who stated being in good or better health were also more concerned about antihypertensive medications than those who reported being in fair or poor health (*p* = 0.001). Of those surveyed, those who lived in the Northern Great Plain (*p* = 0.010) and Southern Great Plain (*p* = 0.049) regions were significantly more concerned about antihypertensive treatment than those who lived in the Pest/Budapest region.

## Discussion

While long-term adherence to antihypertensive therapy remains a critical goal in managing hypertension, this study underscores the importance of patient attitudes as a foundational determinant of medication behavior. Rather than focusing solely on adherence outcomes, it is essential to address the underlying beliefs, perceptions, and emotional responses that shape patients’ willingness to initiate and maintain treatment. Previous research has demonstrated that patients’ beliefs about the necessity of medication and concerns about potential side effects significantly influence adherence patterns [32]. In a systematic review of quantitative studies, Gast and Mathes [33] found that positive health beliefs and perceived benefits were consistently associated with higher adherence rates, suggesting that interventions targeting attitudes may yield more sustainable behavioral change. Moreover, a cross-sectional study conducted in Saudi Arabia revealed that patients with greater knowledge about hypertension and fewer comorbidities were more likely to adhere to prescribed regimens, highlighting the role of education and perceived control [34]. These findings align with the present study’s emphasis on attitudinal factors and suggest that improving patient engagement through tailored communication and shared decision-making may be more effective than adherence-focused strategies alone. Future interventions should therefore prioritize the cultivation of trust, understanding, and empowerment to foster a more receptive attitude toward antihypertensive therapy. This is the first study that describes the simultaneous validation and adaptation of the Hungarian version of the BMQ-General and BMQ-Specific for antihypertensive drug treatment. As the translation of both the BMQ-General and BMQ-Specific for antihypertensive medications showed good reliability and validity, these can be used for research purposes.

A previous research evaluating the Sinhalese translation of the BMQ showed similar values (Cronbach’s alpha: 0.65) regarding the overall internal consistency [35]. In another study evaluating the Portuguese translation of the BMQ the Cronbach’s alpha coefficient for the overall questionnaire was 0.66 [36]. The internal consistency of the Polish BMQ questionnaire among cardiovascular patients was between 0.64 and 0.82 for each of the subscales [37]. The research on BMQ-Specific focusing on cholesterol-lowering medications in Hungary (Necessity: 0.84 and Concern: 0.78) [15] showed very similar values to the current research (Necessity: 0.86 and Concern: 0.85). According to previous research carried out in the Visegrad Group (V4) countries – Czechia, Slovakia, Poland and Hungary –, the Cronbach’s alpha values for Necessity and Concern subscales never went below 0.78. Within the same study, the confirmatory factor analysis showed that the fit of the model was satisfactory for each translation [15]. Overall, both previous and the current studies demonstrate that the BMQ performs well regardless of language or the type of chronic disease.

In the previously mentioned research done among the four Visegrad Group (V4) countries most of the Hungarian respondents belonged to the “indifferent” (31.2%) and “ambivalent” (30.7%) groups. Most of the Czech and Slovak respondents were also in these two groups. Regarding the Polish respondents most of them belonged to the “indifferent” (40.0%) and “ambivalent” (26.5%) groups. The lowest percentage of respondents (10.2% and 11.5%) were “skeptical” about cholesterol-lowering medications [38]. Slightly different results were obtained in the current study, with the difference that most participants belonged to the “indifferent” (38.1%) group, while the second largest group was “accepting” (27.0%). This outcome is the result of the previous research done in the V4 countries. The previous study focused on cholesterol-lowering drugs, while the current study focused on drugs that lower high blood pressure. As this might lead to confusion, we now emphasize this difference in the manuscript.

Thus far, no research had been conducted that specifically analysed attitudes towards antihypertensive medications with a similar statistical approach. Nevertheless, studies focusing on different chronic diseases and with a different methodology have reported that concerns about the adverse effects of medications are independent of patients’ age and education [39]. Therefore, concerns should be addressed regardless of these factors, which is confirmed in the current study as well. Another research done in Sweden concluded that approximately one-third of migraineurs were nonadherent to their medication and belief in medication-related factors could not predict patients’ poor treatment adherence [40]. As these results may suggest that the influence of beliefs on medication adherence depends on the type of chronic disease [41], it is important to explore these influencing factors in relation to all the most commonly used medications using the same measurement tool.

A recent study showed that there is a positive correlation between communication satisfaction and adherence, and that the level of adherence increases with better communication quality [42]. Another research involving Hungarian respondents showed that a patient-centered approach regarding patient-doctor communication is important for efficient and appropriate information exchange [43]. In order to achieve long-term adherence, the above-mentioned two studies highlight the importance that doctors should pay more attention to the quality and style of communication, as they influence the patient’s attitude towards treatment and taking medications.

### Strengths and limitations of the study

One of the strengths of our study was the large sample size (1067 participants), which has the advantage of increasing statistical power. The sample was stratified by age, sex, region and settlement size, increasing the generalizability of the results to the Hungarian hypertensive population. Another strength was that the reliability of the Hungarian translation of the BMQ-General and BMQ-Specific was tested using various statistical methods using appropriate standards.

However, the current study also has some limitations. For example, questions regarding the financial status and health status were self-reported which may have influenced the results of the multivariate analyses. Also, the sample consisted of people who were taking antihypertensive medication, but no information was available on the duration, severity, and possible comorbidities of hypertension. In addition, the data collection took place after the COVID-19 pandemic, which may have influenced participants’ attitudes towards medication [44]. 

Due to data protection regulations and the study design, we were unable to access medical records. Collecting detailed information on comorbidities or other medications would have provided a more comprehensive interpretation.

## Conclusions

Antihypertensive medications are effective in treating the disease when taken correctly. The translation of the BMQ questionnaire is a suitable tool for mapping medication attitudes of Hungarian citizens taking hypertension medications. This tool can be used to identify groups that need more attention when trying to improve attitudes towards antihypertensive medications. The attitude analysis revealed that doctors should pay more attention to patients who are in the “ambivalent” and “skeptical” attitude groups, as these patients are uncertain about taking their medication regularly and these two groups together account for one-third of people taking antihypertensive medication. 

## Data Availability

“Data is provided within the manuscript or supplementary information files”.

## Abbreviations

BMQ: Beliefs About Medicines Questionnaire
CFI: Comparative Fit Index
CI: Confidence interval
COPD: Chronic obstructive pulmonary disease
COVID-19: Coronavirus Disease 2019
L.P.: Limited partnership
Ltd: Limited company
NUTS: Nomenclature of Territorial Units for Statistics
RMSEA: Root Mean Square Error of Approximation
SD: Standard deviation
SRMR: Standardized Root Mean Square Residual
TLI: Tucker-Lewis Index
V4: Visegrad countries
